# A simple microfluidic platform to study age-dependent protein abundance and localization changes in *Saccharomyces cerevisiae*

**DOI:** 10.15698/mic2017.05.573

**Published:** 2017-04-13

**Authors:** Margarita Cabrera, Daniele Novarina, Irina L. Rempel, Liesbeth M. Veenhoff, Michael Chang

**Affiliations:** 1 European Research Institute for the Biology of Ageing, University of Groningen, University Medical Center Groningen, 9713 AV Groningen, the Netherlands

**Keywords:** yeast, replicative ageing, microfluidics, CellASIC, protein abundance, protein localization

## Abstract

The budding yeast *Saccharomyces cerevisiae* divides asymmetrically, with a smaller daughter cell emerging from its larger mother cell. While the daughter lineage is immortal, mother cells age with each cell division and have a finite lifespan. The replicative ageing of the yeast mother cell has been used as a model to study the ageing of mitotically active human cells. Several microfluidic platforms, which use fluid flow to selectively remove daughter cells, have recently been developed that can monitor cell physiology as mother cells age. However, these platforms are not trivial to set up and users often require many hours of training. In this study, we have developed a simple system, which combines a commercially available microfluidic platform (the CellASIC ONIX Microfluidic Platform) and a genetic tool to prevent the proliferation of daughter cells (the Mother Enrichment Program), to monitor protein abundance and localization changes during approximately the first half of the yeast replicative lifespan. We validated our system by observing known age-dependent changes, such as decreased Sir2 abundance, and have identified a protein with a previously unknown age-dependent change in localization.

## INTRODUCTION

With the increasing population of elderly people, it is urgent to focus research on ageing and age-associated diseases. In the last few decades, the budding yeast *Saccharomyces cerevisiae* has been considered a valuable model for understanding longevity in humans 1,2. There are two main models of yeast ageing: replicative and chronological ageing 1,3. Replicative lifespan (RLS) refers to the number of daughter cells produced by a mother cell before death. Replicative ageing is thought to be similar to the ageing of asymmetrically dividing cells in higher eukaryotes (e.g. stem cells). Chronological lifespan is the time a cell survives in a non-dividing state, and is used a model for the ageing of non-proliferating cells like our neurons and muscle cells.

Here, we focus on the study of replicatively ageing yeast cells. The classical method to assay RLS is to manually separate a yeast mother cell away from its new-born daughter cells by micromanipulation 4. A major limitation of this technique is that it is both time and labour intensive. Recently, several microfluidic systems have been developed to allow easier analysis of the RLS of yeast cells 5. A significant advantage of such systems is the ability to monitor cell physiology and fluorescently tagged proteins during the ageing process on the single-cell level. However, the fabrication and use of these microfluidic platforms requires specific infrastructure and training, which is not readily available in most labs. In this study, we have developed a simple and easy-to-use system that exploits a commercially available microfluidic platform (CellASIC) in combination with the Mother Enrichment Program (MEP) 6, which is a genetic tool to prevent the proliferation of daughter cells, to study changes in protein abundance and localization during replicative ageing in yeast. Our system is designed to monitor these changes early during RLS, when cells are still relatively young, because early changes are more likely to have a causative role in replicative ageing, and most characteristics of the ageing phenotype occur early in the yeast lifespan 2. Using our system, we were able to observe several known, and one previously unknown, protein abundance or localization changes that occur during ageing.

## RESULTS 

### Design of a new system to study yeast ageing

**Figure 1:**
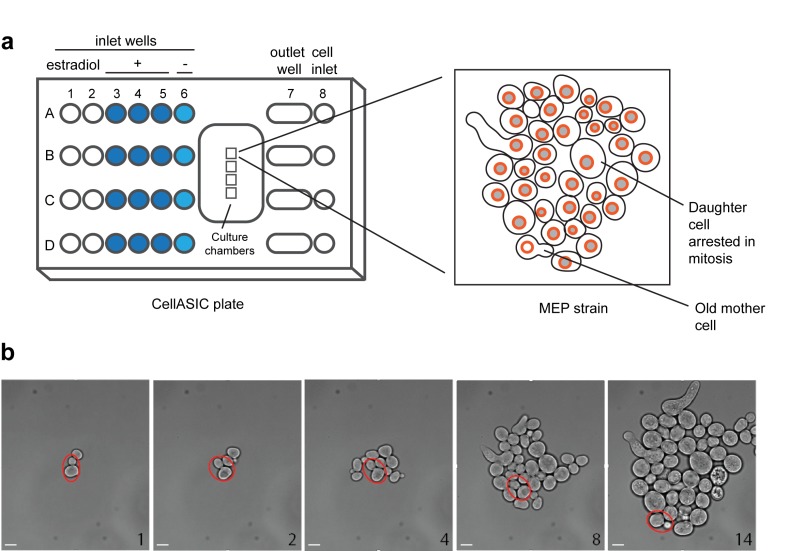
Figure 1: Design of a CellASIC-based platform to study replicative yeast aging. **(a)** Four strains (A-D) can be monitored for long periods using a CellASIC microfluidic system. Once cells are trapped in the culture chambers, perfusion of the media takes place for 60 min and perfusion of the media with estradiol is active for 34 h. After addition of estradiol, only the daughter cells of the MEP strain will be arrested in mitosis and cannot proliferate whereas the mother cell continues dividing. Input wells 1 and 2 are not needed for these experiments and are left empty. **(b)** Bright-field images show the growth of a colony from a founder mother cell (circled in red). Scale bar, 5 µm.

We have developed a simple platform to monitor changes in protein abundance and localization in individual ageing yeast cells. For this purpose, we used the CellASIC ONIX Microfluidic Platform (Merck Millipore) that allows the analysis of four different strains simultaneously (Fig. 1). Although this device maintains cells growing in a flat monolayer, after several divisions it is challenging to follow a single mother cell due to the rapid growth of surrounding cells descended from the mother cell (Movie S1). To circumvent this limitation, we took advantage of the MEP 6, which restricts proliferation of daughter cells after induction with estradiol (Fig. 1, Movie S2). In the MEP system, a Cre recombinase fused to an estradiol-binding domain (Cre-EBD) is expressed from a daughter-specific promoter; introduction of estradiol causes the Cre-EBD to be transported into the nucleus where Cre disrupts two essential genes, *UBC9* and *CDC20*, in daughter cells 6. It has previously been demonstrated that most mother cells live a normal lifespan upon activation of the MEP 6. Consistent with this finding, we found that there is no significant difference in doubling time of wild-type and MEP mother cells grown in the CellASIC device (doubling times: 73 min and 69 min for the wild-type and MEP mother cells observed in Movies S1 and S2, respectively).

We designed a strain (MCY699) so that the genetic components of the MEP can be easily combined with a GFP fusion protein of interest from the Yeast GFP Clone Collection 7 by using Synthetic Genetic Array (SGA) methodology 8. To facilitate analysis of protein localization, we also included Nup49-mCherry as a marker of the nuclear periphery. This strain is available upon request.

### Early changes in protein abundance and/or localization associated with ageing

**Figure 2:**
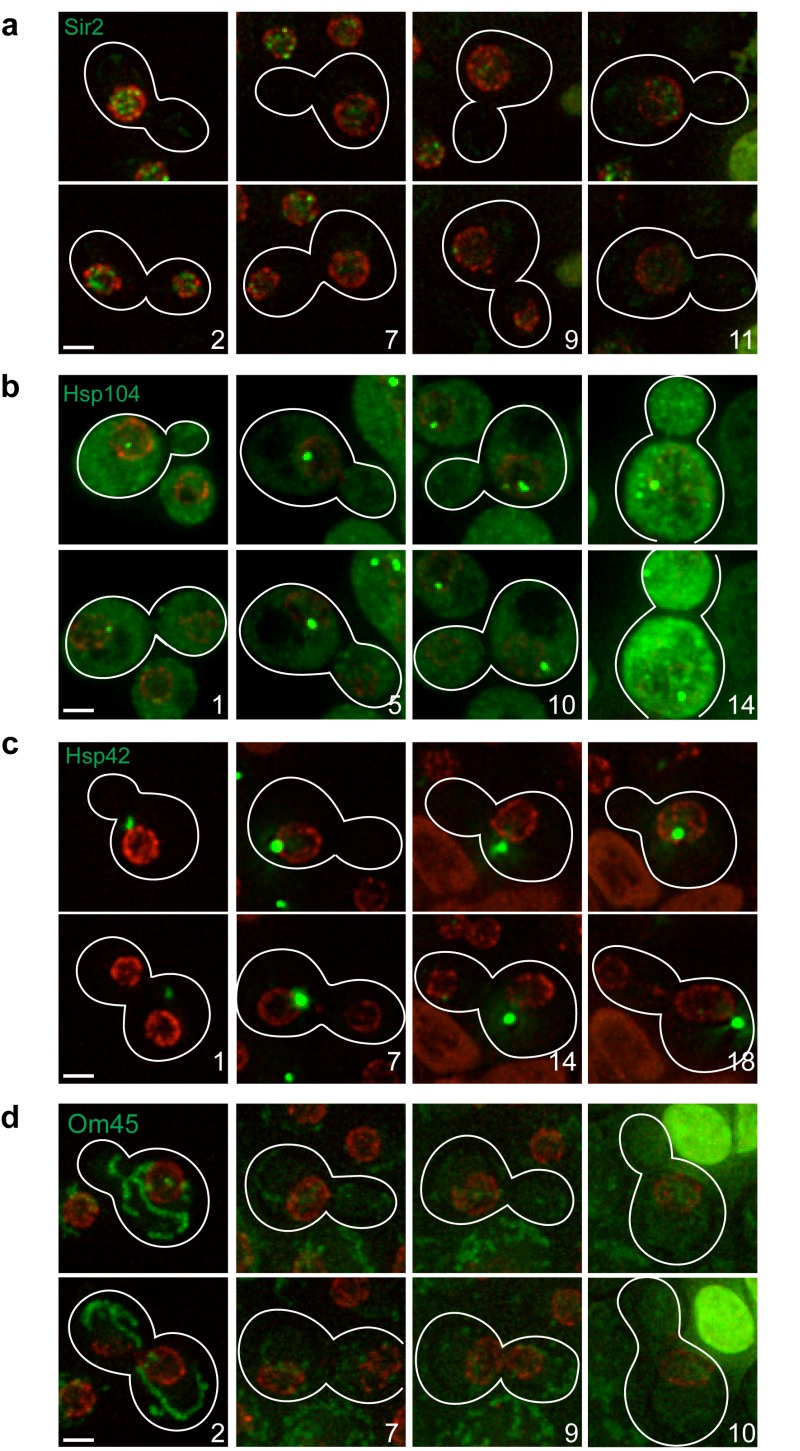
Figure 2: Protein localization changes as cells age. MEP cells expressing Sir2-GFP **(a)**, Hsp104-GFP **(b)**, Hsp42-GFP **(c)**, or Om45-GFP **(d)** were analysed by time-lapse microscopy. Nup49-mCherry was used as marker of the nuclear periphery. Upper and lower images corresponding to the same division, usually 20-60 min apart, are shown to detect possible cell cycle-dependent changes in localization. Stacks of 8-9 sections (0.4 µm spacing) acquired at different time points were deconvolved (maximal projection is shown). Bright-field images were used to draw cell boundaries and count the number of divisions for the mother cells (age indicated with numbers). Representative images are shown. For Sir2-GFP, all six cells analysed showed the same phenotype. For Hsp104-GFP, Hsp42-GFP and Om45-GFP, 10 of 14, 6 of 7 and 11 of 13 cells, respectively, showed the phenotype depicted and described in the text. Scale bar, 2 µm.

To validate our microfluidic-based platform, we examined known changes in protein abundance and/or localization that occur as cells age. The NAD-dependent histone deacetylase Sir2 controls aging by inhibiting recombination at the ribosomal DNA (rDNA) locus 9 and its protein abundance decreases with age 10. In young cells, we find that Sir2 appears as distinct foci by the nuclear periphery, and the intensity of these foci decreases with age (Fig. 2a).

Protein aggregates associate with Hsp104, forming foci that accumulate in number and intensity with age 11. In our experiments, Hsp104 localizes to a single nuclear focus in young cells, and as cells age, the focus becomes larger; towards the end of the time-lapse experiment, cytosolic foci can also be seen, and the overall levels of Hsp104-GFP signal increase (Fig. 2b). This result confirms that protein aggregates cannot be cleared and accumulate in aged cells.

Hsp42 is a protein aggregase responsible for the formation of CytoQ deposits where misfolded proteins are retained in the cytosol 12. Interestingly, an age-associated protein deposit was recently identified, which is distinct from all known stress-induced protein aggregates; Hsp42 is the first protein localizing to this deposit, promoting its formation 13. Accordingly, we find that Hsp42 is found in one cytoplasmic focus in young cells that becomes notably larger as cells age (Fig. 2c). This Hsp42-focus is localized mainly in close proximity to the nuclear periphery.

An early change detected with age is the loss of vacuole acidity that has been shown to trigger mitochondria dysfunction 14. It was reported that mitochondrial membrane potential is reduced in aged cells 14,15, although it is not clear if this event is common to the whole population 16. In agreement with previous results 14, we observed that mitochondria labeled with Om45-GFP fragment show a diffuse staining in old cells in contrast to the typical tubular morphology detectable in young cells (Fig. 2d).

These four examples demonstrate the utility of our microfluidic-based platform to study changes in protein abundance and/or localization during replicative aging.

### Identification of a novel age-dependent change in protein localization

**Figure 3:**
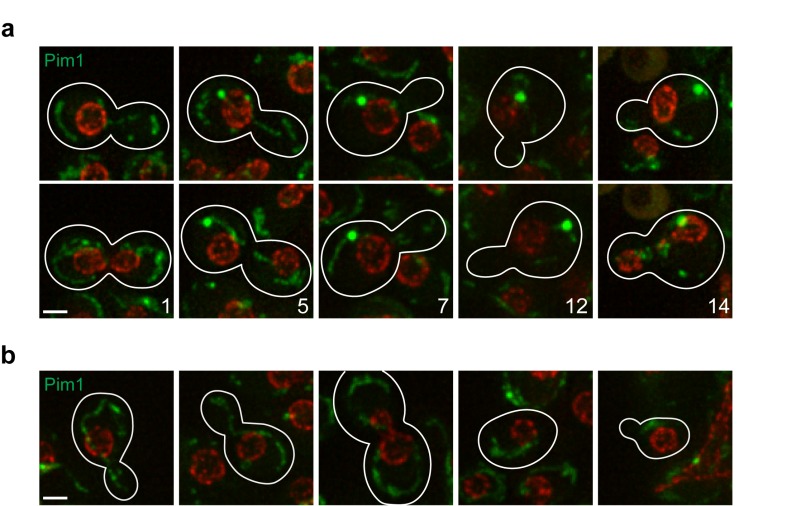
Figure 3: Pim1-GFP localization changes with age. Changes in protein localization were monitored in MEP cells expressing Pim1-GFP **(a, b)**. Nup49-mCherry was used as marker of the nuclear periphery. Upper and lower images corresponding to the same division, but 20-40 min apart, are shown to detect possible cell cycle-dependent changes in localization. Localization of Pim1 in daughter cells **(b)** is shown. Stacks of 4 sections (0.5 µm spacing) acquired at different time points were deconvolved (maximal projection is shown). Bright-field images were used to draw cell boundaries and count the number of divisions for the mother cells (age indicated with numbers). Representative images are shown. All five cells analysed showed the same phenotype depicted and described in the text. Scale bar, 2 µm.

We next examined a panel of proteins whose age-dependent behaviour is not known. Among these, a previously unidentified age-dependent change was observed in the localization of Pim1, which forms a large focus in aged cells (Fig. 3a). The Lon protease Pim1 is required for the removal of mitochondrial damaged proteins 17, so Pim1 might label a new quality control compartment of the mitochondria. Notably, these enlarged Pim1 foci are not present in daughter cells just after division (Fig. 3b), indicating that they are selectively retained in mother cells. Consistent with a link between Pim1 and ageing, it has been shown that *pim1*∆ mutants have a short RLS that can be rescued by overproduction of Hsp104 18. A recent study found that Pim1 is important for a new Hsp104-dependent pathway that sequesters and degrades aggregation-prone proteins in the mitochondria 19. Furthermore, downregulation of Lon promotes cellular senescence in a human melanoma cell line 20.

## DISCUSSION

Microfluidic systems are very powerful tools to examine replicative ageing in yeast 5. This approach allows the monitoring of ageing cells for many generations, and some of the latest designs make the simultaneous analysis of several strains or conditions possible. Importantly, fluorescent markers can be followed throughout the whole lifespan at the single-cell level. One big disadvantage of existing microfluidic systems is that their use often requires special training and equipment and cannot be easily established in every lab 5. In contrast, the CellASIC system is commercially available, easy to handle, and can be connected to any inverted microscope. Moreover, daughter cells in this system are not removed by the media flow and can be analysed together with their progenitor cells. In addition, while most microfluidic systems developed so far for the study of yeast replicative ageing are only compatible with haploid cells 5, the CellASIC system is suitable for both haploid and diploid cells, which is relevant because ploidy can impact RLS 21.

A limitation of our platform is that it works best for analysing changes that appear during the first half of the yeast RLS (i.e. within the first 10-15 divisions) because, even with the MEP preventing the proliferation of daughter cells, the mother cell tends to get surrounded by the non-proliferating daughter cells. Thus, our system cannot be used to measure RLS. However, early changes are more likely to have a causative role in the ageing process. Moreover, most age-dependent changes happen early in the yeast lifespan and get progressively worse with age 2,22.

We were able to reproduce previously published age-dependent changes in protein abundance or localization, thus validating our platform (Fig. 2). We also observed a previously undescribed change in Pim1 localization during replicative ageing (Fig. 3). In aged cells, Pim1 is found in enlarged foci, reminiscent of Tom70 localization, which marks a mitochondrial-derived compartment where the selective degradation of some membrane proteins takes place 16.

A recent tool providing an important contribution to the field of yeast ageing is the MEP 6. In this study, we added new features to this strain by combining the components of the MEP with the components of the SGA methodology, facilitating the potential high-throughput construction of MEP strains that express protein-GFP fusions from the Yeast GFP Clone Collection. Thus, this system could be scaled up to allow the analysis of age-dependent changes in abundance and localization at the proteome-wide level.

## MATERIALS AND METHODS

### Yeast strains and molecular biology

Cells were grown at 30°C in synthetic complete (SC) media 23. The Mother Enrichment Program (MEP) strain (UCC8773 24) was provided by Daniel E. Gottschling (Fred Hutchinson Cancer Research Center, Seattle, WA). The replacement of the *LEU2* marker with *kanMX* in the MEP strain was performed using homologous recombination of PCR fragments 25, creating strain MCY662. This strain was then crossed with strain JTY7 26 (provided by Grant W. Brown, University of Toronto, Canada) by standard yeast genetics to create the strain MCY699. The resulting MEP strain, which also expresses Nup49-mCherry was crossed with the strains from the GFP collection 7 and haploid strains were isolated by sporulation and growth on selective plates.

**Table 1:** Yeast strains used in this study.

Strain name	Relevant genotype	Source
UCC8773	*MAT***a*** his3∆1 leu2∆0 ura3∆0 lys2∆0 ho∆::Pscw11-cre-EDB78-NatMX loxP-CDC20-intron-loxP-HphMX loxP-UBC9-loxP-LEU2*	**24**
JTY7	*MAT*α *NUP49-mCherry::Ca-URA3 can1∆::STE2pr-LEU2 ura3∆0 lyp1∆ leu2∆0 his3∆1 met15∆0*	**26**
MCY662	*MAT***a*** his3∆1 leu2∆0 ura3∆0 lys2∆0 ho∆::Pscw11-cre-EDB78-NatMX loxP-CDC20-intron-loxP-HphMX loxP-UBC9-loxP-kanMX*	This study
MCY699	*MAT*α *his3∆1 leu2∆0 ura3∆0 ho∆::Pscw11-cre-EDB78-NatMX loxP-CDC20-intron-loxP-HphMX loxP-UBC9-loxP-kanMX NUP49-mCherry::Ca-URA3 can1∆::STE2pr-LEU2 lyp1∆*	This study
MCSY22	*MAT***a** *his3∆1 leu2∆0 ura3∆0 ho∆::Pscw11-cre-EDB78-NatMX loxP-CDC20-intron-loxP-HphMX loxP-UBC9-loxP-kanMX NUP49-mCherry::Ca-URA3 can1∆::STE2pr-LEU2 lyp1∆ SIR2-GFP::His3MX6*	This study
MCSY14	*MAT***a** *his3∆1 leu2∆0 ura3∆0 ho∆::Pscw11-cre-EDB78-NatMX loxP-CDC20-intron-loxP-HphMX loxP-UBC9-loxP-kanMX NUP49-mCherry::Ca-URA3 can1∆::STE2pr-LEU2 lyp1∆ HSP104-GFP::His3MX6*	This study
MCSY66	*MAT***a** *his3∆1 leu2∆0 ura3∆0 ho∆::Pscw11-cre-EDB78-NatMX loxP-CDC20-intron-loxP-HphMX loxP-UBC9-loxP-kanMX NUP49-mCherry::Ca-URA3 can1∆::STE2pr-LEU2 lyp1∆ HSP42-GFP::His3MX6*	This study
MCSY17	*MAT***a** *his3∆1 leu2∆0 ura3∆0 ho∆::Pscw11-cre-EDB78-NatMX loxP-CDC20-intron-loxP-HphMX loxP-UBC9-loxP-kanMX NUP49-mCherry::Ca-URA3 can1∆::STE2pr-LEU2 lyp1∆ OM45-GFP::His3MX6*	This study
MCSY63	*MAT***a** *his3∆1 leu2∆0 ura3∆0 ho∆::Pscw11-cre-EDB78-NatMX loxP-CDC20-intron-loxP-HphMX loxP-UBC9-loxP-kanMX NUP49-mCherry::Ca-URA3 can1∆::STE2pr-LEU2 lyp1∆ PIM1-GFP::His3MX6*	This study

### Microfluidics

The microfluidic device CellASIC ONIX Microfluidic Platform (Merck Millipore) was used to maintain yeast cells growing in a monolayer for long periods (Fig. 1). A logarithmically growing culture was diluted to an OD_600_ of 0.1 and 100 µl of cells were placed into a microfluidic plate Y04C. Cells were loaded with a pressure of 6 psi for 5 s. SC media was provided first with a pressure of 5 psi for 5 min to remove the non-trapped cells and then with a pressure of 2 psi for 60 min. Subsequently, SC media containing 1 µM estradiol was applied from three different wells with a total pressure of 2 psi (flow rate of 10 µl/h) for 34 h (Figure 1). For each time-lapse experiment, we analysed four strains that show similar expression level of the corresponding GFP-proteins and selected 20 individual cells per strain.

### Fluorescence microscopy

Images were acquired using a Deltavision Elite imaging system (Applied Precision (GE), Issaquah, WA, USA) composed of an inverted microscope (IX-71; Olympus) equipped with an PLAPON 60X (1.4 NA) oil immersion objective, InsightSSITM Solid State Illumination, excitation and emission filters for FITC and A594, image-based autofocus, ultimate focus and a coolSNAP HQ2 camera (Photometrics, Tucson, AZ, USA). Time-lapse images were acquired with an interval of 20 min for a total time of 35 h. The exposure time for the FITC channel ranges between 20 and 100 ms. With an exposure time of 100 ms, fluorescent images were acquired every 20 min in the time-point intervals 1-10 (i.e. 20 min time point to 200 min time point), 42-50, 72-80 and 102-110. A bright-field image acquired at each time point was used to quantify the number of divisions (age). Stacks of 4-9 images with 0.4-0.5 µm spacing were subjected to 3D deconvolution using SoftWoRx 5.5 software (Applied Precision). Processing of all images was performed using Fiji (ImageJ, National Institute of Health).

## SUPPLEMENTAL MATERIAL

Click here for Movie 1.

Click here for Movie 2.

All supplemental data for this article are also available online at http://microbialcell.com/researcharticles/a-simple-microfluidic-platform-to-study-age-dependent-protein-abundance-and-localization-changes-in-saccharomyces-cerevisiae/.
